# Postnatal Testicular Activity in Healthy Boys and Boys With Cryptorchidism

**DOI:** 10.3389/fendo.2019.00489

**Published:** 2019-07-23

**Authors:** Tanja Kuiri-Hänninen, Jaakko Koskenniemi, Leo Dunkel, Jorma Toppari, Ulla Sankilampi

**Affiliations:** ^1^Department of Pediatrics, Kuopio University Hospital, Kuopio, Finland; ^2^Research Centre for Integrative Physiology and Pharmacology, Institute of Biomedicine, University of Turku, Turku, Finland; ^3^Department of Pediatrics, Turku University Hospital, Turku, Finland; ^4^Barts and the London, William Harvey Research Institute, Queen Mary University of London, London, United Kingdom

**Keywords:** HPG axis, cryptorchidism, minipuberty of infancy, testicular descent, gonadotropin (FSH and LH)

## Abstract

Cryptorchidism, or undescended testis, is a well-known risk factor for testicular cancer and impaired semen quality in adulthood, conditions which have their origins in early fetal and postnatal life. In human pregnancy, the interplay of testicular and placental hormones as well as local regulatory factors and control by the hypothalamic-pituitary (HP) axis, lead to testicular descent by term. The normal masculine development may be disrupted by environmental factors or genetic defects and result in undescended testes. Minipuberty refers to the postnatal re-activation of the HP-testicular (T) axis after birth. During the first weeks of life, gonadotropin levels increase, followed by activation and proliferation of testicular Leydig, Sertoli and germ cells. Consequent rise in testosterone levels results in penile growth during the first months of life. Testicular size increases and testicular descent continues until three to five months of age. Insufficient HPT axis activation (e.g., hypogonadotropic hypogonadism) is often associated with undescended testis and therefore minipuberty is considered an important phase in the normal male reproductive development. Minipuberty provides a unique window of opportunity for the early evaluation of HPT axis function during early infancy. For cryptorchid boys, hormonal evaluation during minipuberty may give a hint of the underlying etiology and aid in the evaluation of the later risk of HPT axis dysfunction and impaired fertility. The aim of this review is to summarize the current knowledge of the role of minipuberty in testicular development and descent.

## Introduction

Testicular descent initiates during early fetal development and finalizes during the first postnatal months. This complex process is regulated by multiple genetic, anatomical and hormonal factors and environmental factors may influence its course. Failed testicular descent (i.e., cryptorchidism) is one of the most common congenital anomalies with prevalence between 2 and 9% (1, 2). However, the etiology of isolated cryptorchidism often remains unknown.

**Figure 1:**
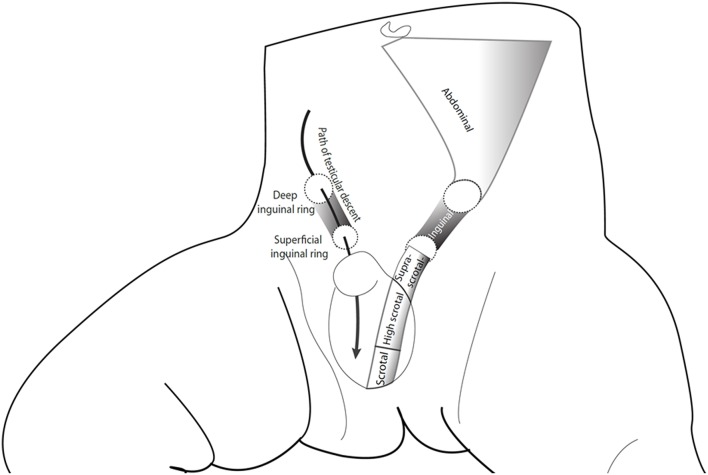
The path of testicular descent and classification of testicular position according to the John Radcliffe Hospital Cryptorchidism Study Group (5). The figure is reproduced from Koskenniemi (6) with the permission of the copyright holder.

Definitions related to congenital cryptorchidism vary. In his landmark paper, Scorer considered testes that were within the 4 cm distance from the pubic bone as undescended among term boys (3), which roughly corresponds with the 2.5th centile at birth (4). However, most cohort studies have used criteria developed by John Radcliffe Hospital Study group, in which the testicular position is classified in reference to anatomical landmarks as “non-palpable,” “inguinal,” “suprascrotal,” and “high scrotal,” whereas testes that lie in the bottom of the scrotum (scrotal) are considered normal (Figure 1) (5). Testes that can be manipulated to the bottom of the scrotum, and stay there at least for a while are termed retractile and are often considered normal (7). However, data on long-term fertility and testicular cancer morbidity outcomes within this subgroup of cryptorchidism remain scarce. Some authors have questioned whether high scrotal testes indeed should be considered abnormal (8). On the other hand, there seems to be some evidence of reduced testicular growth among boys with retractile testes (9).

**Figure 2:**
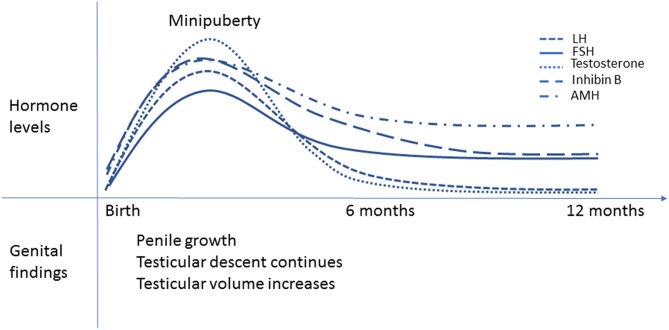
Minipuberty of infancy. Schematic presentation of the changes in reproductive hormone levels during the first year of life in healthy boys. The peak hormone levels are observed between 1-3 months of age. LH and testosterone levels decrease by 6 months of age, but FSH and inhibin B levels remain elevated longer. AMH levels increase from birth to 3 months of age, then slightly decrease but remain higher than in adults until puberty. Penile length and testicular volume increase and testicular descent continues during minipuberty.

Complete testicular descent requires adequate and timely function of the fetal Leydig cells from the first to the third trimester of pregnancy. Factors that interfere with Leydig cell function may predispose infants to cryptorchidism. During the first trimester, placental hCG regulates Leydig cell function. During the second trimester, the fetal hypothalamic-pituitary (HP) unit takes control. Besides the critical intrauterine phases of testicular development, the early postnatal period has emerged as an important period in male reproductive development. The HPT axis is transiently activated during the first months of life (10–12) and this activation is associated with penile and testicular growth as well as continued testicular descent (4, 12, 13). The minipuberty of infancy is transient since HPT axis activity decreases toward the 6 month of postnatal life (12) (Figure 2).

In milder cases of cryptorchidism, spontaneous testicular descent is common during minipuberty, but rarely seen thereafter (14, 15). There are reports of infants with congenital hypogonadotropic hypogonadism (CHH) and absent minipuberty who present with testicular ascent and involution of the penis and scrotum during the first year of life (16). Moreover, two small studies indicated that among boys with cryptorchidism due to hypogonadotropic hypogonadism, gonadotropin therapy induces testicular descent and surgical orchidopexy may be avoided (17, 18). However, CHH is a very rare cause of cryptorchidism and treatment with human chorionic gonadotropic hormone (hCG) or GnRH analogs is no longer recommended, mainly because of poor efficacy and possible adverse effects (19, 20).

In this review, we evaluate and summarize the current knowledge of the role of the HPT axis in pre- and postnatal testicular descent in humans and compare the differences in minipuberty between healthy and cryptorchid boys.

## Overview and Ontogeny of the HPT Axis

Specific gonadotropin-releasing hormone (GnRH) secreting neurons in the hypothalamus are the major controllers of the reproductive function. During early embryogenesis, GnRH neurons migrate from the nasal placode to their final location in the anterior hypothalamus and complete their journey by 15 weeks gestation (21, 22). The function of the mature GnRH neurons is modulated by several other hypothalamic hormones (23), such as glutamate and γ-aminobutyric acid (GABA), and many neuropeptides, including neuropeptide Y, galanin-like peptide, opioid peptides, and orexins. However, the most important upstream regulator of the GnRH neurons is a neuromodulatory peptide kisspeptin, encoded by the *KISS1* gene and acting via a G protein-coupled receptor GPR54 that is now termed KISS1R (24). Kisspeptin neurons can co-express neurokinin B and dynorphin, termed kisspeptin-neurokinin B-dynorphin (KNDy) neurons (25). Currently, the ontogenesis of this complex neuronal network in humans is not fully understood.

Pulsatile release of GnRH results in pituitary secretion of two gonadotropins, luteinizing hormone (LH) and follicle stimulating hormone (FSH). Both LH and FSH are detectable in the fetal pituitary and peripheral circulation by 12–14 weeks gestation (26–28). LH and FSH bind to their specific receptors in the testicular Leydig and Sertoli cells, respectively. Androgens secreted by the Leydig cells and inhibin B secreted by the Sertoli cells regulate the activity of the HP unit by negative feedback. The negative feedback effects of testosterone are largely mediated by estradiol after local aromatization of testosterone to estradiol in the brain. Inhibin B specifically regulates pituitary FSH secretion, whereas the negative feedback by estradiol suppresses the secretion of both LH and FSH (29).

During the first trimester of pregnancy, testicular hormone secretion is not dependent on the integrity of the HP unit of the fetus. Instead, Leydig cells are stimulated by placental hCG which acts through the LH/hCG receptor. Leydig cell activity is critical for fetal masculinization programming during a discrete window between 8 and 14 weeks gestation (30). Notably, defects in HP function do not affect masculinization as the axis is still immature at this time. In addition to the canonical steroidogenic pathway in the testis, there is an alternative backdoor pathway of androgen production in the fetus that uses the placenta and liver in androgen synthesis (31). Disruption of either pathways lead to problems in masculinization (32).

The fetal HP unit is functional during the second trimester and testicular testosterone and inhibin B are major regulators. The activity of the fetal HPT axis peaks during the late first and early second trimester when testosterone levels reach levels observed in adult men (33). At this time, fetal gonadotropin levels markedly differ between sexes as LH and FSH levels are higher in female fetuses (27, 28). This sex difference is probably due to the negative feedback effects on the fetal HP unit mediated by the testicular testosterone and inhibin B. In males, inhibin B levels are higher compared to levels observed in female fetuses at midgestation and levels remain elevated until term (34). The activity of the HPT axis decreases toward the end of gestation and is low at term. This shift is possibly due to maturing negative feedback mechanisms and suppression mediated by increased placental hormone levels, especially estrogens (34, 35).

### The Role of Fetal HPT Axis Activity in Intrauterine Testicular Development and Descent

By 6 weeks of gestation, the bipotential gonad differentiates into the testes and by week 8, Leydig cells produce testosterone and Sertoli cells produce anti-Müllerian hormone (AMH). This early hormonal activity guides the normal development of the internal and external male reproductive organs (36, 37). Initial testicular development occurs proximal to the posterior abdominal wall. The abdominal phase of the testicular descent begins between the 7th cervical and 8th thoracic vertebra with the initial formation of the gonadal streak (38). From that position, the testes descend to the 9th thoracic to 3rd lumbar vertebra during the first months post-conception. This initial descent is caused by the descent of the primordium of the diaphragm and the regression of the mesonephros. A ligament called *Gubernaculum Hunteri* anchors the testis to the abdominal wall at developing opening of the inguinal canal. Swelling of the gubernaculum during gestational week 20 dilates the inguinal canal and the gubernaculum migrates down the inguinal canal (39). This transinguinal testicular descent usually occurs between gestational weeks 23–30 (39, 40).

Leydig cell product, insulin-like 3 (INSL3) and its receptor RXFP2 play a central role in the transabdominal phase of testicular descent—which is usually completed by 15 weeks of gestation (41, 42). In mice with *Insl3* inactivation, swelling of the gubernaculum does not occur, and testicular descent is terminated high in the abdomen (43). In humans, mutations in *INSL3* and *RXFP2* are rare, but have been associated with cryptorchidism (44). Human fetal INSL3 secretion is regulated by hCG and LH *in vitro* (45). Unexpectedly, a study found that cryptorchid cases appeared to have increased INSL3 levels in the amniotic fluid compared to controls between gestational weeks 13–16 (43). INLS3 seemed lower in cryptorchid fetuses vs. controls during gestational weeks 17–22, suggesting altered Leydig cell activity later when the testicular descent continues, although the difference was below the level of statistical significance, possibly due to larger variability in INSL3 concentrations (46).

The role of androgens in the first phase of testicular descent is not completely understood. However, inguinoscrotal testicular descent is generally thought to be androgen dependent (42, 47). Complete lack of androgen activity in 46, XY fetuses with inactivating mutations of the androgen receptor (AR) results in phenotypic female development and abdominal/inguinal testis. Milder forms result in a range of phenotypes including micropenis, cryptorchidism, bifid scrotum and/or hypospadias (48). Similar phenotypes may result from mutations in the LH/hCG receptor (*LHR*), which block androgen synthesis in the testis (49). Fetal Leydig cell control transitions from placental hCG to pituitary LH somewhere during late first to early second trimester of pregnancy. Low circulating hCG levels have been observed during gestational weeks 12–16 in mothers of boys with cryptorchidism at birth (50). During the inguinoscrotal phase of testicular descent, hCG levels decline and pituitary LH is important for stimulating Leydig cell androgen production. In fetuses with CHH and very low LH levels, testosterone levels during the second and third trimester are low, often resulting in micropenis and cryptorchidism (51, 52).

In addition to INSL3 and androgens, AMH may play a role in testicular descent. Indeed, there is a high prevalence of cryptorchidism among subjects with mutations in *AMH* or *AMHR2* (i.e., persistent Müllerian duct syndrome) (53). However, the exact mechanism is unclear as *Amh* knock-out mice have normally descended testes (54).

INSL3 levels in the cord blood of cryptorchid boys are reduced compared with boys with normally descended testes (55, 56). Cryptorchid boys with a non-palpable testis have lower cord INSL3 levels compared to boys with palpable testes (56). Notably, levels of testosterone, LH, FSH, AMH, sex hormone binding globulin (SHBG) and inhibin B in cord blood do not differ between cryptorchid boys and controls (56).

### Postnatal Activation of the HPT Axis, Minipuberty

Near term, activity of the fetal HPT axis is low. Cord blood testosterone and inhibin B levels are higher in boys than in girls indicating that the testes are not completely inactive (34, 57). hCG levels remain relatively high in the cord blood, but hCG, in addition to the high placenta-derived estrogen levels, are cleared from the newborn's circulation during the first week of life. Removal of the suppressive effects of placental hormones after birth results in re-activation of the newborn's HP unit as demonstrated by increased LH and FSH levels during the first weeks of life (12, 58–60). This gonadotropin surge results in testicular activation and consequent increase in testosterone (11, 12, 61), inhibin B (11, 62), and AMH levels (60, 63).

#### Gonadotrope (LH)-Leydig Cell Axis

Testosterone levels increase concomitantly with LH levels until 1–3 months of life. Serum levels reach the pubertal range and then decline toward the age of 6 months (11, 61, 62). In boys, LH levels exceed FSH levels during the first postnatal months. As pituitary activity wanes, LH levels fall below FSH levels around 3 months of age (62). By 6–9 months of age, LH and testosterone levels decline and remain at low levels for the duration of the childhood (11, 12). INSL3 levels correlate positively with both LH and testosterone in boys at the age of 3 months, suggesting that postnatal LH also stimulates INSL3 secretion (55). INSL3 levels are higher in infancy than later in childhood (55) and increase again at puberty (64).

The biological activity of testosterone during minipuberty has been questioned as SHBG levels increase simultaneously—leading to low levels of free testosterone (65). However, observations from longitudinal studies suggest the opposite effect. The testosterone surge in minipuberty is associated with simultaneous biological effects in androgen target tissues as evidenced by penile growth, transient secretion of prostate specific antigen and sebaceous gland activity (12, 13, 66). Surging testosterone levels are also associated with accelerated growth velocity during minipuberty and probably explain why boys grow faster than girls in early infancy (67). Moreover, in boys who lack minipuberty because of CHH, linear growth in infancy is slower compared to healthy boys (68). Postnatal testosterone levels potentially modulate the masculine neurobehavioral development, as testosterone levels during minipuberty have been associated with male-typical play behavior at 14 months of age (69). In another study, penile growth during minipuberty was associated with sex-typed play behavior at 3–4 years of age (70). Testosterone levels in minipuberty have been associated with early language development (71).

#### Gonadotrope (FSH)-Sertoli Cell Axis

In newborn boys, both inhibin B and AMH levels increase and peak near the age of 3 months. Inhibin B levels at this time exceed adult levels and AMH levels reach their highest level of the entire lifespan (11, 62, 63, 72). Inhibin B levels at 3 months of age correlate negatively with FSH levels (62, 73). Unlike LH levels, FSH levels often remain measurable during childhood (11). Inhibin B levels decline at approximately 15 months of age falling to the prepubertal range then increase again during puberty (11, 72). AMH levels decline toward 1 year of age, but remain high during childhood until increased testosterone levels in puberty cause AMH level to decline (63).

Notably, testicular volume increases during minipuberty (12, 73). This growth most likely represents the proliferation of the Sertoli cells in the seminiferous compartment (74, 75). Furthermore, the number of Leydig and germ cells increase during the first postnatal months (74–76). Testicular volume and inhibin B correlate positively at 3 months of age (73). The AR is not present in Sertoli cells in fetal or newborn testis. This observation likely explains why high intratesticular testosterone levels do not activate spermatogenesis and why AMH levels remain high (77, 78). Following the minipubertal gonadotropin surge, testicular volume appears to slightly decrease during infancy (12, 79). In addition to testicular growth, testicular descent continues after birth. Recent data from a large prospective Danish-Finnish birth cohort study suggest that the testes clearly migrate toward the bottom of the scrotum between birth and 3 months of age (4). Testicular position is associated with indices of both Leydig and Sertoli cell function (i.e., testosterone:LH and inhibin B:FSH ratios) as well as IGF-I levels (4). Furthermore, in cryptorchid and non-cryptorchid boys, a small but significant testicular ascent is observed between 3 and 18 months of age (4). This fits well with the observations that spontaneous testicular descent is unlikely after minipuberty (15, 80).

In preterm boys born before 37 weeks of gestation, cryptorchidism is a common finding. However, spontaneous testicular descent usually occurs by term or during the first months of postnatal life. Indeed, testicular descent after 3 months of age is more common in preterm than in full-term boys (14). The prolonged period of spontaneous testicular descent probably reflects the longer period of postnatal HPT axis activity in preterm boys (12). However, when corrected for the age of prematurity, HPT activity wanes at a developmental age similar to full-term boys (12). Consequently, the duration of minipuberty seems to be programmed according to the child's developmental age.

#### Germ Cells

There are limited human data on the early development of germ cells and related regulatory factors (81). In a post-mortem study of testicular pathology samples from 48 boys aged <3 years, the number of germ cells was higher in boys between the ages of 50–150 days (~2–5months-old) compared to either younger or older boys (74). In another post-mortem study, germ cell apoptotic index (i.e., percentage of apoptotic cells/total cell number) increased and proliferation index decreased after the first month of life (*n* = 18) whereas no further change was observed between boys 1 and 6 months of age (*n* = 13) and 1–6 years of age (*n* = 13) (82). Gonocytes, the fetal stem cells, mature during the first year of life into adult dark (Ad) spermatogonia, that are presumed to be the stem cells for later spermatogenesis (83). This maturation coincides with minipuberty. Accordingly, a causal role for hormonal activity during minipuberty has been suggested (84). However, the role of gonadotropins and testicular hormones in this process has yet to be clarified and existing data on the role of androgens are controversial (85). The maturation of spermatogonia continues after minipuberty and some primary spermatocytes can be observed around 3–4 years of age (86).

Consequently, minipuberty is important for male reproductive development as it seems to finalize and stabilize the genital development of boys. It is clear that testicular hormones exert negative feedback on the HP unit in infancy. However, as agonadal children exhibit a biphasic pattern of gonadotropin levels (87), the factors that silence HP unit activity during childhood do not appear to be of gonadal origin.

### Minipuberty in Cryptorchid Boys

Available data on reproductive hormone levels in cryptorchid boys during minipuberty are limited and interpreting the existing data is challenging due to the small number of patients in most studies, variation in the age at sampling and classification of testicular position and the phenotypes (e.g., unilateral/bilateral, palpable/non-palpable, etc). As hormone levels during the first months of life undergo developmental changes (described above), interpreting values observed in cryptorchid infants must be done with caution. In contrast, boys with anorchia present a typical hormonal profile during minipuberty (and childhood) with very high LH and FSH levels and low or undetectable levels of testosterone, inhibin B and AMH—regarded as the most specific marker of existing testicular tissue (87). Studies on association of reproductive hormone levels and testicular position in infancy are summarized in Table 1.

**Table 1:** Studies reporting the association between reproductive hormone levels and testicular position in infant boys.

**References**	**Participants**	**Age at hormonal measurements**	**Outcome measures**	**Results**
Gendrel et al. (88)	27 transiently cryptorchid, 30 persistently cryptorchid	1–4 months	LH, FSH, T	Lower LH and T levels in persistently cryptorchid vs. transiently cryptorchid boys.
De Muinck Keizer-Schrama et al. (89)	160 control, 19 transiently cryptorchid, 29 persistently cryptorchid	1) 3, 6, 12 months 2) 12 months	1) basal and LHRH-stimulated LH and FSH, basal T 2) hCG-stimulated T	Higher basal LH levels in transiently cryptorchid than in controls. No difference in T levels between the groups.
Raivio et al. (90)	35 control, 45 cryptorchid	3 months	Serum androgen bioactivity	Quantifiable serum androgen bioactivity in 46% of boys with scrotal/high scrotal testicular position, but not in any of the boys with suprascrotal or higher testicular position.
Barthold et al. (91)	26 control, 20 cryptorchid	2 months (plasma), serial urine samples up to 4 months	Plasma LH, FSH, T, E2, inhibin B, leptin, SHBG; urine LH, FSH, T, E2	No differences between controls vs. cryptorchid boys.
Suomi et al. (92)	Finnish boys: 300 control, 88 cryptorchid Danish boys: 399 control, 34 cryptorchid	3 months	LH, FSH, T, inhibin B	Higher FSH levels in cryptorchid than in control boys. Lower Inhibin B and higher LH in Finnish (not in Danish) cryptorchid boys vs. controls. No difference between groups in T levels.
Bay et al. (55)	100 control, 28 transiently cryptorchid, 51 persistently cryptorchid	Birth,3 months	INSL3, LH, T	Cord blood INSL3 reduced in persistently vs. transiently cryptorchid boys and controls. LH to INSL3 ratio higher in persistently vs. transiently cryptorchid boys at 3 months of age.
Pierik et al. (93)	113 control, 43 cryptorchid	1-6 months	LH, FSH, T, AMH, inhibin B, SHBG	Lower T and non-SHBG-bound T in cryptorchid than in control boys
Fenichel et al. (56)	128 control, 52 cryptorchid	Birth	INSL3, T, LH, FSH, AMH, hCG, SHBG	Lower INSL3 levels in cryptorchid vs. control boys. No difference in other hormone levels.
Koskenniemi et al. (4)	2,545 boys Blood tests: Finnish 362, Danish 680	3 months	Testicular distance to pubic bone at birth, 3 and 18 months; LH, FSH, T, INSL3, inhibin B	Testosterone/LH-ratio and inhibin B/FSH-ratio were positively associated with lower testicular position.
Grinspon et al. (94)	1) 24 control, 19 cryptorchid (7 bilateral) 2) 26 control, 80 cryptorchid (52 bilateral)	1) 1–5.9 months 2) 6 months- 1.9 years	LH, FSH, T, AMH	1) Lower T in unilaterally cryptorchid vs. control boys. 2) Lower AMH levels in bilaterally vs. unilaterally cryptorchid boys and controls.

#### Gonadotrope (LH)-Leydig Cell Axis in Cryptorchidism

Higher minipuberty LH levels in cryptorchid boys compared to boys with normally descended testes have been reported in some (92), but not all studies (89, 91, 93). Gendrel et al. reported higher LH and T levels in cryptorchid boys with spontaneous testicular descent compared to boys who remained cryptorchid during minipuberty (88).

Despite the central role of androgens in testicular descent, circulating concentrations of testosterone seem to be comparable between congenitally cryptorchid and non-cryptorchid boys at birth (56). In addition, comparing testosterone levels in cryptorchid and non-cryptorchid boys at the age of two to 3 months does not reveal significant differences between the groups (91, 92).

However, testicular position at 3 months (or the change in testicular position between birth and 3 months) may be related to reproductive hormone levels during minipuberty. A small Finnish study noted the differences in circulating testosterone levels between cryptorchid and healthy boys at 3 months (90). Furthermore, Raivio et al. (90) found measurable androgen bioactivity at the age of 3 months in 26 of 64 (41%) of boys with scrotal or high scrotal testes, whereas androgen bioactivity was undetectable in all 16 boys with suprascrotal/inguinal/non-palpable testes.

In a Finnish-Danish cohort study, higher testosterone levels were observed among boys who were cryptorchid at birth yet underwent spontaneous testicular descent at 3 months compared to healthy controls (92). Boys with mild cryptorchidism (i.e., high scrotal testis) at the age of 3 months also exhibited higher testosterone levels than those with severe cryptorchidism (suprascrotal, inguinal or non-palpable) (92). A similar difference in testosterone levels between cryptorchid boys with spontaneous descent and those who remained cryptorchid was noted in an Egyptian study (80). Likewise, Job et al. found higher testosterone levels during minipuberty in boys with spontaneous descent than those who remained cryptorchid (88, 95). Finally, a Dutch study reported an overall difference in the testosterone levels between cryptorchid and healthy boys and revealed that a higher proportion of cryptorchid boys had undetectable testosterone levels compared to controls at 100+ days postnatal (93). These observations are in contrast to older data from a Dutch study of similar gonadotropins (i.e., basal and stimulated) and testosterone levels in cryptorchid compared to control boys at 3, 6, or 12 months of age (89).

In the longitudinal Finnish-Danish cohort study, the testosterone:LH-ratio correlated positively with improved testicular position from birth to 3 months in cryptorchid and non-cryptorchid boys alike (4). These observations suggest that persisting cryptorchidism through minipuberty might reflect a deficiency in androgen action. Indeed, reduced Leydig cell numbers have been reported in testicular biopsies of cryptorchid boys compared with those with normally descended testes during the first months of life (83).

At 3 months of age, a higher LH to INSL3 ratio has been reported among persistently cryptorchid boys compared to healthy boys. Further, INSL3 correlated with LH, testosterone and inhibin B in healthy boys yet no correlations were found in cryptorchid boys (55).

Data on biological effects of testosterone in cryptorchid boys in infancy are scarce. In one study at birth, cryptorchidism was associated with shorter penile length yet penile length was not adjusted for gestational age—which was lower among the cryptorchid boys. At 3-months follow up, no differences were observed (15).

#### Gonadotrope (FSH)-Sertoli Cell Axis in Cryptorchidism

Higher FSH levels have been reported in cryptorchid 3-month-olds compared to healthy boys (92). In several smaller studies, no differences were observed (88, 89, 91, 93).

Lower inhibin B levels have been reported in a cohort of Finnish cryptorchid boys than in healthy boys at 3 months of age whereas among Danish boys, no significant difference was observed (92). Similarly, Pierik et al. found no differences in inhibin B or AMH levels during minipuberty when comparing cryptorchid boys to controls (93).

While gonadotropin and testosterone levels decline to very low levels by 6 months of age, inhibin B and AMH levels remain measurable after minipuberty. At 2 years of age, inhibin B and AMH levels were lower in cryptorchid boys prior to treatment (*n* = 27) compared to controls (*n* = 27) (96). Further, boys with bilateral cryptorchidism have even lower levels of inhibin B and AMH compared to unilateral cases. In boys aged 6 months to 8.9 years, Grinspon et al. reported lower AMH levels in boys with bilateral cryptorchidism (*n* = 186) vs. controls (*n* = 179). However, AMH levels did not differ between unilateral cases (*n* = 124) and controls (94). In principle, these results may partially reflect the germ cell/Sertoli cell damage as a consequence of abnormal testicular location.

Testicular volume in cryptorchid boys reflects the number of Sertoli and germ cells (97). In unilateral cryptorchidism, the maldescended testis is smaller after birth and grows slower during the first 6 months of life compared to the contralateral scrotal testis (98). In a randomized controlled trial, early surgery at 9 months of age (vs. later orchidopexy) resulted in larger testicular volume at 3 years of age (97, 99). Neither gonadotropin, testosterone nor inhibin B levels measured during minipuberty (at 2 months of age) predicted the number of Sertoli or germ cells (97).

#### Germ Cell Development in Cryptorchidism

After 1–2 years of age, the number of germ cells in cryptorchid testes is lower than in normally descended testis (97, 100, 101). These observations form the rationale for the early surgical treatment between 6-12 months. Early maturation of germ cells has been shown to be diminished and delayed in cryptorchid testis (84). In boys with unilateral cryptorchidism who are younger than 1 year old, disappearance of gonocytes is delayed and the number of Ad spermatogonia is diminished in the undescended testis compared to the contralateral descended (scrotal) testis (100). The number of Ad spermatogonia in pre-operative testicular biopsy (prior to 9 months of age) seems to be associated with future sperm count as all boys with Ad spermatogonia at orchidopexy subsequently have normal sperm counts—in contrast to abnormally low sperm counts in those boys with no Ad spermatogonia (102).

### Changes in Testicular Position After Minipuberty

A longitudinal study in England revealed that 4% of 12-month-old boys had acquired cryptorchidism (i.e., testes that ascended from the scrotum to suprascrotal, inguinal or abdominal position during infancy) (15). Later during childhood, the prevalence decreased (0.6–1.3%) (103). A cross-sectional Dutch study suggests that the prevalence of acquired cryptorchidism is approximately 1.1–2.2% in childhood (104). Further, a small but significant testicular ascent of approximately 5 mm was observed between the age of three and 18 months in a prospective longitudinal study (4).

The prognosis of acquired cryptorchidism is not well-characterized. In the majority of cases, testes descend during mid-puberty (105, 106). Despite the tendency for spontaneous descent, semen quality is reduced compared to controls—and comparable to levels observed in congenital cryptorchidism (107). Some authors speculate that the risk of malignancy might be lower in acquired than in congenital cryptorchidism (53). However, this has not yet been definitively proven. Most clinical guidelines advocate operative treatment (i.e., orchidopexy) in the absence of randomized controlled studies to indicate otherwise (19, 20, 108).

Little is known about the etiology of the testicular ascent. A British study showed that up to 40 percent of boys with congenital cryptorchidism and spontaneous testicular descent during minipuberty were noted to have a cryptorchid testis again at the age of 12 months (5). Thus, it has been proposed that slight abnormalities in prenatal testicular descent (i.e., borderline cases of congenital cryptorchidism) might later manifest as acquired cryptorchidism during growth in childhood (109). Recently published data indicate that in contrast to overall body growth, the testes physiologically descend during and subsequently ascend after minipuberty (4). Interestingly, the lack of sufficient Leydig cell action during minipuberty may be associated with acquired cryptorchidism. Compared to controls, slower penile growth has been noted during minipuberty among boys who later exhibited acquired cryptorchidism (15). In addition, two retrospective studies observed an association between acquired cryptorchidism and hypospadias (110, 111)—which has been thought to be caused by reduced androgen action.

Thus, we regard it plausible that altered minipuberty might be associated with the pathogenesis of acquired cryptorchidism.

### Management of Cryptorchidism

Cryptorchidism is associated with impaired fertility and an increased risk of developing germ cell cancer (112, 113) and hypogonadism (114). Early surgical treatment has been shown to improve fertility, but the effect on cancer risk remains controversial (115, 116). The amount of the lost germ cells in testicular biopsies at the time of orchidopexy has been associated with the timing of the surgical treatment (97, 117) and recent guidelines advise early treatment between 6 and 12 months, and at the latest, by 18 months of age. However, surgery is often delayed beyond this age. In a survey conducted in Germany and Switzerland between 2009 and 2015, 81% of boys with congenital cryptorchidism underwent orchidopexy after 1 year and 54% after the age of 2 years often because of a late referral (118).

Despite the early surgical repair, some cryptorchid boys become infertile. Bilateral disease and non-palpable position are associated with worse fertility outcomes. Hormonal treatment with hCG injections (or intranasal GnRH) is no longer recommended due to the lack of evidence of long-term efficacy and possible adverse effects (19, 20). Currently, there are ongoing studies examining the effectiveness of adjuvant GnRH analog treatment with orchidopexy with the intention of maturing spermatogenic stem cells as maturation is presumed (but not proven) to be associated with HPT axis activity during normal minipuberty (119, 120).

### Hormonal Treatment of Cryptorchidism in Congenital Hypogonadotropic Hypogonadism (CHH)

But for cases of CHH, hormonal treatment of cryptorchidism is not recommended (19, 20). The efficacy of hormonal treatment for cryptorchidism is poor and has potential side effects as interstitial bleeding, inflammation, and germ cell apoptosis (121–123).

Approximately 30% of boys with CHH have undescended testis. Cases are equally distributed between unilateral and bilateral cryptorchidism (51, 52). Cryptorchidism is more common than micropenis (29 vs. 15%). In a Finnish-Danish cohort of 36 boys with CHH, history of cryptorchidism and/or micropenis was reported in half of the patients (68). Acquired cryptorchidism has also been described in CHH. Main et al. reported three cases of hypogonadotropic hypogonadism and early acquired cryptorchidism in association with penile involution (16). All patients responded to testosterone therapy.

Lambert and Bougneres reported effects of combined recombinant LH and FSH treatment to mimic minipuberty in boys < 12 months old with CHH (isolated *n* = 5, combined *n* = 3) (17). At treatment onset, five boys had non-palpable testes and three had high scrotal testes. Duration of treatment was ~6 months and cost ~$18,000 per child. All boys had testicular descent during the treatment and only one boy had re-ascent (at 11 months of age, requiring orchiopexy). Very recently, Papadimitriou et al. reported results of three-month replacement therapy with subcutaneous recombinant LH/FSH for 10 boys with CHH and associated micropenis and cryptorchidism beginning from the median age of 0.35 years (18). The treatment resulted in high normal and supranormal LH and FSH levels, respectively, and normal testosterone and inhibin B levels for age. Penile length increased and testicular descent to scrotal position took place in all boys, however two required orchidopexy later. During the follow-up of 3–10 years scrotal testicular position has been maintained for all cases. At the moment, there are no data on long-term effects of this treatment on later fertility.

## Conclusions

Minipuberty appears to be an important phase in finalizing and stabilizing of the normal male genital development. Minipuberty is associated with penile and testicular growth as well as testicular descent. Moreover, proliferation and maturation of Sertoli and germ cells during minipuberty are seemingly important for later fertility potential. Some boys with cryptorchidism undergo spontaneous testicular descent during minipuberty but are at increased risk of later re-ascent, and a careful follow-up is warranted. Notably, spontaneous descent is rare after the first 3–6 months of age.

Despite the central role of HPT axis in testicular descent, reproductive hormone levels in cryptorchid infant boys are often in the normal range for age. This might reflect the multifactorial, yet poorly understood, etiology of cryptorchidism. In certain pathologic situations, hormone levels in cryptorchid boys during minipuberty may present a recognizable pattern. These include anorchia together with very high gonadotropin levels and low/undetectable testosterone, AMH and inhibin B levels, or CHH presenting with cryptorchidism and small, but normally formed penis (i.e., micropenis) in the setting of very low gonadotropin and testosterone levels. Based on available data, higher gonadotropin and lower inhibin B levels during minipuberty in some cryptorchid boys may reflect a primary testicular defect/dysfunction. However, it is not known whether these changes are the cause or merely a consequence of the testicular maldescent.

Based on the evidence documented in this review, the following are presented as guidelines for management of cases of cryptorchidism before the age of 6 months and referral for orchidopexy. Infants with normal penile size and anatomy and no family history of reproductive disorders do not need hormonal evaluation for isolated unilateral cryptorchidism. However, boys with bilateral non-palpable testes, palpable testes but other signs of a possible disorders/differences of sex development (DSD) (i.e., severe hypospadias, bifid scrotum) and boys with associated micropenis should immediately be referred for a pediatric endocrine consultation. Despite careful investigation, the etiology remains unknown in most cases of cryptorchidism. However, in boys with bilateral cryptorchidism or micro-orchidism, hormonal evaluation during minipuberty can be useful. Evaluation should include LH, FSH, and testosterone measurement at 1–2 months of age while AMH and inhibin B level may be informative later. Hormonal treatment of cryptorchidism should be considered by a pediatric endocrinologist only in those cases with an identified hormonal defect such as in CHH or partial androgen insensitivity.

## Author Contributions

All authors listed have made a substantial, direct and intellectual contribution to the work, and approved it for publication.

### Conflict of Interest Statement

The authors declare that the research was conducted in the absence of any commercial or financial relationships that could be construed as a potential conflict of interest.
